# Deep Learning Analysis Using ^18^F-FDG PET/CT to Predict Occult Lymph Node Metastasis in Patients With Clinical N0 Lung Adenocarcinoma

**DOI:** 10.3389/fonc.2022.915871

**Published:** 2022-07-07

**Authors:** Ming-li Ouyang, Rui-xuan Zheng, Yi-ran Wang, Zi-yi Zuo, Liu-dan Gu, Yu-qian Tian, Yu-guo Wei, Xiao-ying Huang, Kun Tang, Liang-xing Wang

**Affiliations:** ^1^ Key Laboratory of Heart and Lung, Division of Pulmonary Medicine, The First Affiliated Hospital of Wenzhou Medical University, Wenzhou, China; ^2^ Department of Medical Engineering, The First Affiliated Hospital of Wenzhou Medical University, Wenzhou, China; ^3^ Precision Health Institution, General Electric (GE) Healthcare, Hangzhou, China; ^4^ Department of Nuclear Medicine, The First Affiliated Hospital of Wenzhou Medical University, Wenzhou, China

**Keywords:** positron emission tomography/computed tomography (PET/CT), convolutional neural network, lung adenocarcinoma, sublobar resection, lymph node status

## Abstract

**Introduction:**

The aim of this work was to determine the feasibility of using a deep learning approach to predict occult lymph node metastasis (OLM) based on preoperative FDG-PET/CT images in patients with clinical node-negative (cN0) lung adenocarcinoma.

**Materials and Methods:**

Dataset 1 (for training and internal validation) included 376 consecutive patients with cN0 lung adenocarcinoma from our hospital between May 2012 and May 2021. Dataset 2 (for prospective test) used 58 consecutive patients with cN0 lung adenocarcinoma from June 2021 to February 2022 at the same center. Three deep learning models: PET alone, CT alone, and combined model, were developed for the prediction of OLM. The performance of the models was evaluated on internal validation and prospective test in terms of accuracy, sensitivity, specificity, and areas under the receiver operating characteristic curve (AUCs).

**Results:**

The combined model incorporating PET and CT showed the best performance, achieved an AUC of 0.81 [95% confidence interval (CI): 0.61, 1.00] in the prediction of OLM in internal validation set (n = 60) and an AUC of 0.87 (95% CI: 0.75, 0.99) in the prospective test set (n = 58). The model achieved 87.50% sensitivity, 80.00% specificity, and 81.00% accuracy in the internal validation set and achieved 75.00% sensitivity, 88.46% specificity, and 86.60% accuracy in the prospective test set.

**Conclusion:**

This study presented a deep learning approach to enable the prediction of occult nodal involvement based on the PET/CT images before surgery in cN0 lung adenocarcinoma, which would help clinicians select patients who would be suitable for sublobar resection.

## Introduction

Lung cancer is one of the most common malignancies and the leading cause of death from cancer worldwide (1). Lung adenocarcinoma (LUAD) is the most common histologic subtype of lung cancer (2). Currently, lobectomy with systemic nodal dissection is the standard treatment for patients with early-stage non–small cell lung cancer (NSCLC) (3), and recently, limited surgery (wedge resection or segmentectomy) has also been performed to preserve healthy lung tissue (4–6). Accurate staging to confirm node-negative (N0) status is required for limited surgery. If N0 status is unreliable, then lobectomy with systemic nodal dissection rather than limited surgery is mandatory. ^18^F-fluorodeoxyglucose positron emission tomography/computed tomography (^18^F-FDG PET/CT) is a valuable imaging modality for evaluation of lymph node (LN) or distant metastasis of lung cancers. Although PET/CT is more sensitive to assess LN status than traditional examinations, occult LN metastasis (OLM) still occurs at a high rate (14%–21%) (7–9). The definition for OLM was that clinical N0 (cN0) staged by PET/CT was pathologically confirmed LN metastasis (LNM) after surgery. Thus, there is a strong need to develop reliable non-invasive methods to identify patients with OLM from cN0 patients staged by PET/CT.

In recent years, radiomics has received increasing attention, and it is a technique for high-throughput extraction of quantitative features from medical images (10, 11). Indeed, many studies have exhibited that quantitative radiomic image features of the primary tumor could be used as non-invasive biomarkers to predict LNM and were good predictive performance (12–14). For OLM of LUAD, Zhong et al. (15) reported that the radiomics signature of the primary tumor based on CT scans had a significant predictive value. Our previous research (16) found that a PET-based radiomics model had achieved success in the prediction of OLM in patients with LUAD. However, traditional radiomic methods are based on four time-consuming and complex steps (tumor segmentation, feature extraction, feature selection, and modeling). Moreover, observer-dependent differences may cause poor repeatability in case of manual segmentation.

Deep learning is a new and especially promising approach that automatically learns powerful feature representations from images, text, or sound and has been shown to sometimes surpass human-level performance in task-specific applications (17–19). Compared with the conventional radiomic methods, the deep learning method simplifies the analysis process and avoids subjective bias because it does not require VOI definition or segmentation. More recently, the deep learning method using convolutional neural network (CNN) has been widely applied to analyze medical images and has been effective in diseases detection and classification (20–22). For classifying LN, Zhao et al. (23) developed a cross-modal deep learning system based on CT images to accurately predict LN metastasis in stage T1 LUADs. Tau et al. (24) reported that using a CNN to analyze PET images can yield a reasonably good prediction of nodal metastasis in patients with NSCLC. In view of the fact that previous studies predicted LN metastasis using deep learning, we hypothesized that deep learning based on PET/CT images might play an important role in predicting OLM.

Hence, the purpose of this study was to evaluate the capability of deep learning analysis based on a two-dimensional (2D) CNN architecture for the prediction of OLM through the use of preoperative FDG-PET/CT images of cN0 LUAD.

## Materials and Methods

### Patients

A total of 434 patients (193 men and 241 women) with cN0 LUAD who had pretreatment FDG PET/CT and underwent surgical resection with the systematic LN dissection from May 2012 to February 2022 were enrolled in this study at The First Affiliated Hospital of Wenzhou Medical University. Among these patients, 343 (79.0%) were pN0 after surgery and pathological examination. In other words, the prevalence of OLM with PET/CT was 21.0% in LUAD, which is basically consistent with previous studies (7–9). The criteria for cN0 on PET/CT was all LNs’ short-axis diameter of less than 10 mm without FDG uptake higher than the surrounding background (25). The interval between PET/CT scan and surgery was shorter than 3 weeks in all patients. The exclusion criteria for patients were as follows: (I) history of other malignancy; (II) distant metastasis; (III) multiple lesions; (IV) neoadjuvant chemotherapy/radiotherapy; (V) images with poor quality due to the leakage of ^18^F-FDG at the injection site, low signal-to-noise ratio, respiratory artifacts, and other movement artifacts. Staging was performed according to the eighth edition of the Union for International Cancer Control TNM classification.

**Figure 1:**
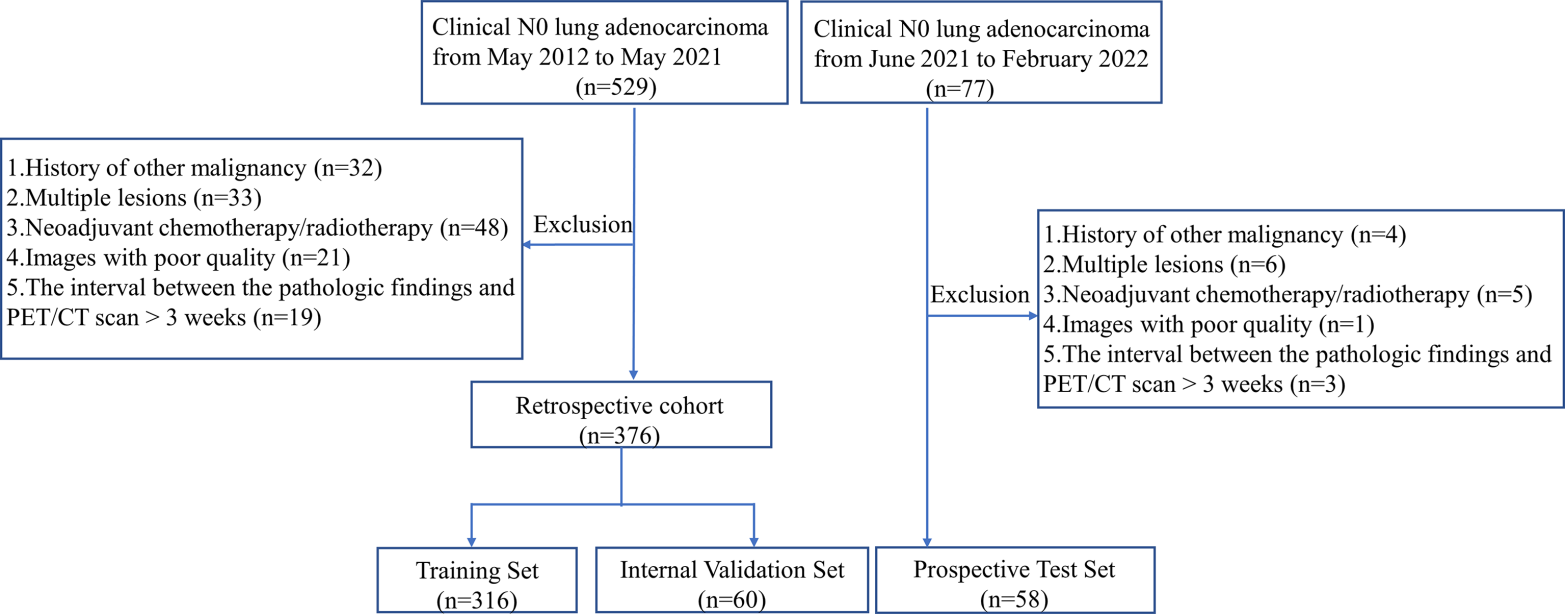
The flowchart of the patient selection.

Dataset 1 included 376 consecutive patients from our Hospital between May 2012 and May 2021. Dataset 2 used 58 consecutive patients from June 2021 to February 2022 at the same center. Sixty patients from dataset 1 were randomly allocated to the internal validation dataset, and the remaining 316 patients were assigned to the training set. Dataset 2 was taken as an independent set for the prospective test. The prospective test is a more powerful method for evaluating the model performance than random splitting of a single set or cross-validation because it allows for non-random variation between sets (26). A flowchart of patient selection is shown in **Figure 1**.

This study was approved by the Institutional Review Board of our hospital. Informed consent from the retrospective patients was waived, and written informed consent was provided for patients in prospective test set.

### PET/CT Acquisition

An integrated PET/CT scanner (GEMINI TF 64; Philips, The Netherlands) was used for all patients. At least 6-h fasting and serum glucose levels below 110 ml/dl were required before being injected with ^18^F-FDG (3.7 MBq/kg). Sixty minutes after intravenous injection, the body was scanned in the supine position. A low-dose unenhanced CT scan from skull base to the middle thighs was obtained with the following parameters: 120 kV, 80 mA, pitch of 0.829, and reconstruction thickness and interval of 5.0 mm. After CT completion, PET images were acquired by using the 3D model with the following parameters: field of view of 576 mm, a matrix of 144 × 144, slice thickness and interval of 5.0 mm, and an emission scan time of each bed position of 1.5 min. PET images were iteratively reconstructed by the ordered subset expectation maximization algorithm, using CT image for attenuation correction.

### Image Selection and Processing

FDG uptake at the primary tumor site was identified on PET images with reference to the CT part of PET-CT. Reconstruction in the sagittal and coronal planes was done from the axial images. Slices with the largest tumor area were selected in axial, coronal, and sagittal planes of PET and CT images. To reduce the computational expense and improve model’s accuracy, all selected images were cropped to contain only the entire chest as much as possible. Then, the images were converted from the Digital Imaging and Communications in Medicine to Joint Photographic Experts Group format pictures. Subsequently, we resized the images to 299 × 299 pixels and normalized the pixel values to a range of 0 to 1.

There was a higher frequency of OLM negative (OLMN). To overcome the imbalance problem between the two groups (positive or negative), we applied three times oversampling for positive samples and two times oversampling for negative samples to ensure the ratio of the two groups near 1:1. Furthermore, image augmentation, including image rotation and flipping for total of four times, was performed on the training dataset.

### CNN Model Architecture

**Figure 2:**
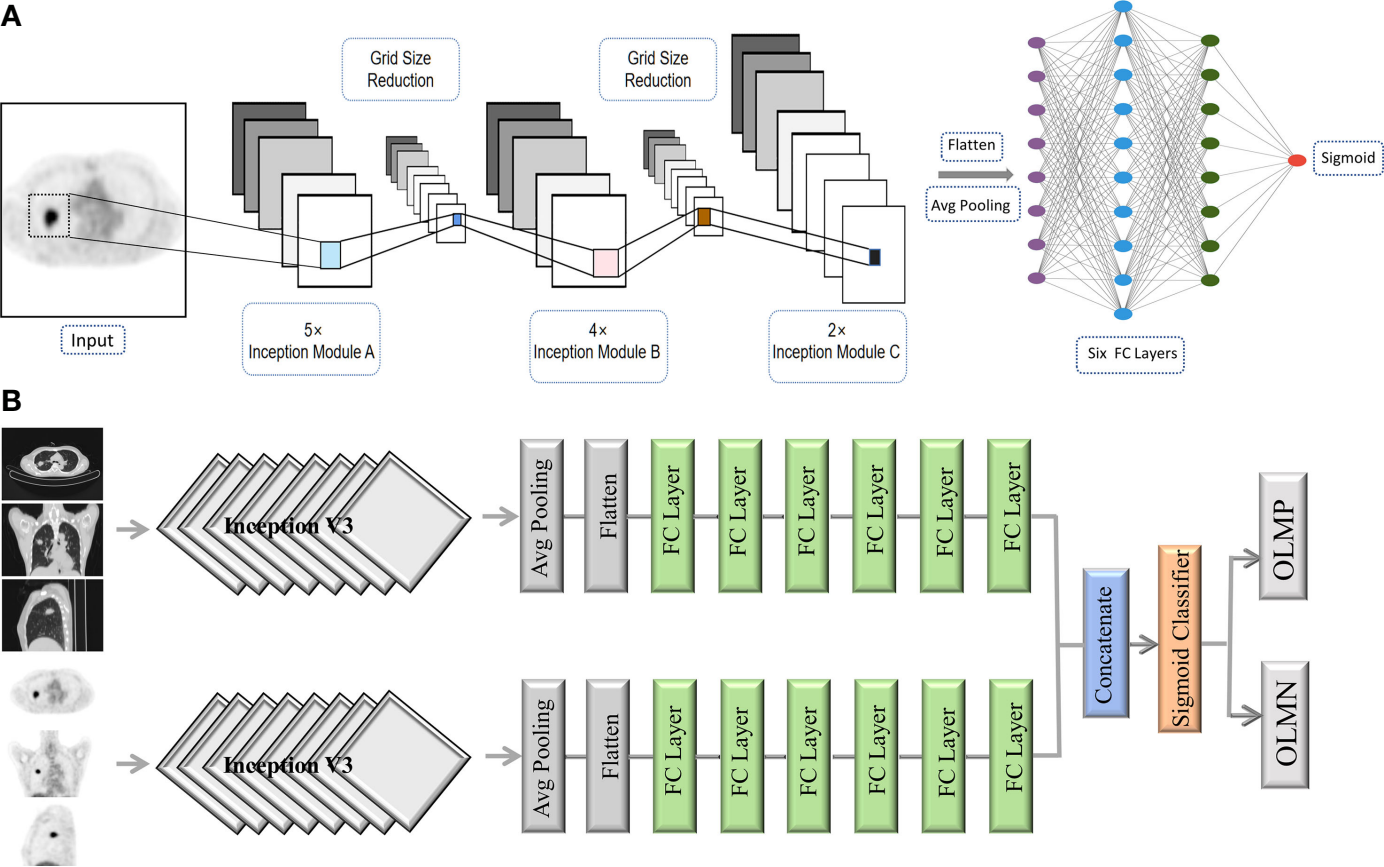
The architecture of the CNN **(A)**. Schematic overview of the combined model (PET + CT) **(B)**. Avg pooling, average pooling; FC layer, fully connected layer; OLMN, occult lymph node metastasis negative; OLMP, occult lymph node metastasis positive.

Respective model (PET or CT): The deep CNN model used was the Inception V3 architecture in this study (27). Transfer learning was applied using weights pretrained on the ImageNet dataset. We arranged three channels (299 × 299 × 3 pixels) in the input layer. Three 2D slices (axial, coronal, and sagittal) were used as input to the CNN network rather than 3D volume data because 2D-based analysis enabled us to reduce GPU memory usage and limit the overfitting. The generated features from the Inception V3 were flattened into a 1D feature vector after the average pooling layer. In the end, six fully connected layers and a sigmoid layer were connected to enable the classification of OLMN and OLM positive (OLMP). To avoid overfitting, dropouts were used. The architecture of the CNN is shown in **Figure 2A**.

Combined model (PET + CT): For the construction of complex model, PET and CT were first respectively run to the last full connect layer, then combined them together, and finally connected a sigmoid layer (1 nodes) for classification. Schematic overview of the combined model is shown in **Figure 2B**.

All the above analyses were implemented in the Keras library in Python, using TensorFlow as backend (Python 2.7, Keras 2.6.0, TensorFlow 2.6.0). Adam with a learning rate of 0.000012 and a batch size of 32 was used for parameters optimization. The number of epochs of training was set to 100.

### Model Performance

For assessing the performance of prediction models, the receiver operating characteristic (ROC) curves were displayed in the training, internal validation, and prospective test sets, respectively. The performance metrics such as accuracy, sensitivity, specificity, and the area under the curve (AUC) were calculated. Fivefold cross-validation was used to verify the generalization ability.

### Statistical Analysis

The statistical analyses were implemented by using IBM SPSS (version 25.0) and Python (version 2.7). Categorical data were analyzed with the chi-square test and the Fisher’s exact test. Numerical data were analyzed with the unpaired t-test, Mann–Whitney U-test, ANOVA, and Kruskal–Wallis test. For missing data, mode imputation was used for categorical variables, and mean imputation was used for continuous variables. P-values less than 0.05 indicated a statistically significant difference.

## Results

### Baseline Information

The baseline patient characteristics are shown in **Table 1**. The sample sizes of the training, internal validation, and prospective test sets were 316, 60, and 58, respectively. No statistical differences, including age (p = 0.663), gender (p =0.820), smoking history (p = 0.418), tumor location (p = 0.522), radiologic lesion type (p= 0.244), tumor SUVmax (p = 0.261), carcinoembryonic antigen (CEA) (p = 0.250), and predominant subtype (p = 0.088), among the three sets were observed except for pathologic tumor size (p = 0.011) in **Table 1**.

**Table 1:** Baseline characteristics of datasets.

Characteristics	Training Set	Internal Validation Set	Prospective Test Set	P-Value
	(n = 316)	(n = 60)	(n = 58)	
Age (years) ^*^	62.29 ± 9.73	63.17 ± 9.44	63.36 ± 11.83	0.663
Sex				0.820
Female	178 (56.3)	33 (55.0)	30 (51.7)	
Male	138 (43.7)	27 (45.0)	28 (48.3)	
Smoking history				0.418
Ever smoker	78 (24.7)	16 (26.7)	10 (17.2)	
Never smoker	238 (75.3)	44 (73.3)	48 (82.8)	
Tumor location				0.522
RUL	97 (30.7)	14 (23.4)	19 (32.8)	
RML	20 (6.3)	6 (10.0)	8 (13.8)	
RLL	70 (22.2)	11 (18.3)	10 (17.2)	
LUL	81 (25.6)	18 (30.0)	14 (24.1)	
LLL	48 (15.2)	11 (18.3)	7 (12.1)	
Radiologic lesion type				0.244
Pure solid	288 (91.1)	53 (88.3)	49 (84.5)	
Subsolid	28 (8.9)	7 (11.7)	9 (15.5)	
Tumor SUVmax^*^	5.62 ± 3.59	4.63 ± 2.43	5.70 ± 4.34	0.261
CEA, ng/ml^*^	7.76 ± 33.89	4.06 ± 2.73	6.75 ± 11.82	0.25
Pathologic tumor size^*^	23.31 ± 10.36	19.87 ± 8.83	23.64 ± 9.93	0.011
Predominant subtype				0.088
Acinar	232 (73.4)	41 (68.3)	42 (72.4)	
Papillary	34 (10.8)	6 (10.0)	9 (15.6)	
Lepidic	25 (7.9)	4 (6.7)	0 (0)	
Solid	13 (4.1)	4 (6.7)	6 (10.3)	
Micropapillary	1 (0.3)	1 (1.6)	0 (0)	
Colloid	11 (3.5)	4 (6.7)	1 (1.7)	

RUL, right upper lobe; RML, right middle lobe; RLL, right lower lobe; LUL, left upper lobe; LLL, left lower lobe; CEA, carcinoembryonic antigen.

*Data are means ± standard deviations.

### Comparison of Clinicopathologic Data Between OLMN and OLMP Groups

A comparison of clinicopathologic data between OLMN and OLMP groups in the three sets is presented in **Table 2**. OLMP was identified in 91 of all 434 patients (20.9%). The training set of 316 patients included 75 OLMP (23.7%) and 241 OLMN (76.3%). The internal validation set of 60 patients included 8 OLMP (13.3%) and 52 OLMN (86.7%). The prospective test set of 58 patients included 8 OLMP (13.8%) and 50 OLMN (86.2%). Detailed information about the distribution of N stages for OLMP cases of three datasets is shown in **Table 2**. In addition, similar tendencies were observed for pathologic tumor size, CEA, and tumor SUVmax, respectively, in the three sets, although not always statistically significant.

**Table 2:** Comparison of clinical features between OLMN and OLMP groups in the three sets.

Characteristics	Training Set			Internal Validation Set			Prospective Test Set		
	(OLMN = 241; OLMP = 75)			(OLMN = 52; OLMP = 8)			(OLMN = 50; OLMP = 8)		
	OLMN	OLMP	P	OLMN	OLMP	P	OLMN	OLMP	P
Age (years) ^*^	63.03 ± 9.46	59.91 ± 10.24	0.015	63.29 ± 9.44	62.38 ± 10.01	0.801	63.22 ± 11.70	64.25 ± 13.48	0.822
Sex			0.125			0.939			0.299
Female	130 (53.9)	48 (64.0)		28 (53.8)	5 (62.5)		24 (48.0)	6 (75.0)	
Male	111 (46.1)	27 (36.0)		24 (46.2)	3 (37.5)		26 (52.0)	2 (25.0)	
Smoking history			0.004			1			0.375
Ever smoker	69 (28.6)	9 (12.0)		14 (26.9)	2 (25.0)		10 (20.0)	0 (0)	
Never smoker	172 (71.4)	66 (88.0)		38 (73.1)	6 (75.0)		40 (80.0)	8 (100)	
Tumor location			0.650			0.736			0.597
RUL	76 (31.5)	21 (28.0)		13 (25.0)	1 (12.5)		16 (32.0)	3 (37.5)	
RML	14 (5.8)	6 (8.0)		6 (11.5)	0 (0)		8 (16.0)	0 (0)	
RLL	52 (21.6)	18 (24.0)		10 (19.2)	1 (12.5)		8 (16.0)	2 (25.0)	
LUL	65 (27.0)	16 (21.3)		14 (26.9)	4 (50.0)		11 (22.0)	3 (37.5)	
LLL	34 (14.1)	14 (18.7)		9 (17.4)	2 (25.0)		7 (14.0)	0 (0)	
Radiologic lesion type			0.031			0.608			0.436
Pure solid	215 (89.2)	73 (97.3)		45 (86.5)	8 (100)		41 (82.0)	8 (100)	
Subsolid	26 (10.8)	2 (2.7)		7 (13.5)	0 (0)		9 (18.0)	0 (0)	
Tumor SUVmax^*^	4.96 ± 3.24	7.74 ± 3.85	< 0.001	4.45 ± 2.40	5.82 ± 2.42	0.064	5.22 ± 4.36	8.64 ± 2.95	0.002
CEA, ng/mL^*^	5.62 ± 9.15	15.24 ± 67.42	0.029	3.11 ± 2.19	4.5 ± 2.46	0.046	4.64 ± 2.88	19.18 ± 29.52	0.311
Pathologic tumor size^*^	22.18 ± 9.62	26.93 ± 11.78	< 0.001	19.23 ± 8.82	24.00 ± 8.25	0.056	21.24 ± 7.19	38.63 ± 11.94	< 0.001
Predominant subtype			0.318			0.399			0.629
Acinar	171 (71.0)	61 (81.3)		36 (69.2)	5 (62.5)		37 (74.0)	5 (62.5)	
Papillary	26 (10.8)	8 (10.7)		5 (9.6)	1 (12.5)		7 (14.0)	2 (25.0)	
Lepidic	23 (9.5)	2 (2.6)		4 (7.7)	0 (0)		0 (0)	0 (0)	
Solid	10 (4.1)	3 (4.0)		2 (3.9)	2 (25.0)		5 (10.0)	1 (12.5)	
Micropapillary	1 (0.4)	0 (0)		1 (1.9)	0 (0)		0 (0)	0 (0)	
Colloid	10 (4.2)	1 (1.4)		4 (7.7)	0 (0)		1 (2.0)	0 (0)	
pN (8th ed.)									
N1a (single N1)		31 (41.3)			3 (37.5)			3 (37.5)	
N1b (multiple N1)		6 (8.0)			0 (0)			0 (0)	
N2a (single N2)		17 (22.7)			2 (25.0)			0 (0)	
N2b (multiple N2)		21 (28.0)			3 (37.5)			5 (62.5)	

OLMN, occult lymph node metastasis negative; OLMP, occult lymph node metastasis positive; RUL, right upper lobe; RML,

right middle lobe; RLL, right lower lobe; LUL, left upper lobe; LLL, left lower lobe; CEA, carcinoembryonic antigen.

*Data are means ± standard deviations.

### Performance of Deep Learning Models

**Figure 3:**
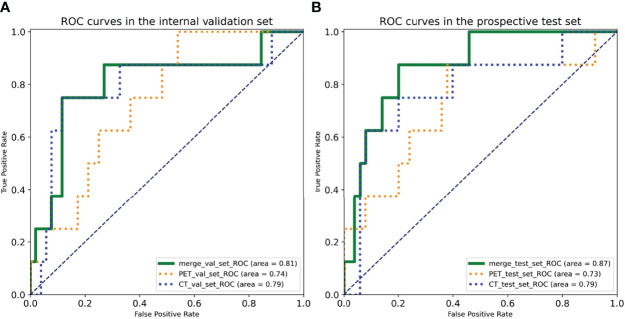
Receiver operating characteristic (ROC) curves of three deep learning models in the **(A)** internal validation set and the **(B)** prospective test set.

The deep learning models demonstrated good predictive performance for OLM with the use of the primary lung cancer images of internal validation set, with AUCs of 0.74 [95% confidence interval (CI): 0.58, 0.90) for the PET model, 0.79 (95% CI: 0.58, 1.00) for the CT model, and 0.81 (95% CI: 0.61, 1.00) for the complex model. For prospective test set, the AUCs were 0.73 (95% CI: 0.51, 0.95) for the PET model, 0.79 (95% CI: 0.59, 0.98) for the CT model, and 0.87 (95% CI: 0.75, 0.99) for the complex model (**Figure 3**). The discriminatory ability of the complex model displayed the highest in the validation and test sets.

For internal validation set, the sensitivities of PET, CT, and combined models were 75.00%, 75.00%, and 87.50%, respectively; the specificities of PET, CT, and combined models were 63.46%, 88.46%, and 80.00%, respectively; and the accuracies of PET, CT, and combined models were 65.00%, 86.67%, and 81.00%, respectively (**Table 3**).

**Table 3:** Performance of the three deep learning models.

	PET			CT			Combined		
	Sensitivity (%)	Specificity (%)	Accuracy (%)	Sensitivity (%)	Specificity (%)	Accuracy (%)	Sensitivity (%)	Specificity (%)	Accuracy (%)
Internal Validation Set	75.00%	63.46%	65.00%	75.00%	88.46%	86.67%	87.50%	80.00%	81.00%
Prospective Test Set	87.50%	62.00%	65.52%	75.00%	80.00%	79.31%	75.00%	88.46%	86.60%

For prospective test set, the sensitivities of PET, CT, and combined models were 87.50%, 75.00%, and 75.00%, respectively; the specificities of PET, CT, and combined models were 62.00%, 80.00% and 88.46%, respectively; and the accuracies of PET, CT, and combined models were 65.52%,79.31% and 86.60%, respectively (**Table 3**).

**Figure 4:**
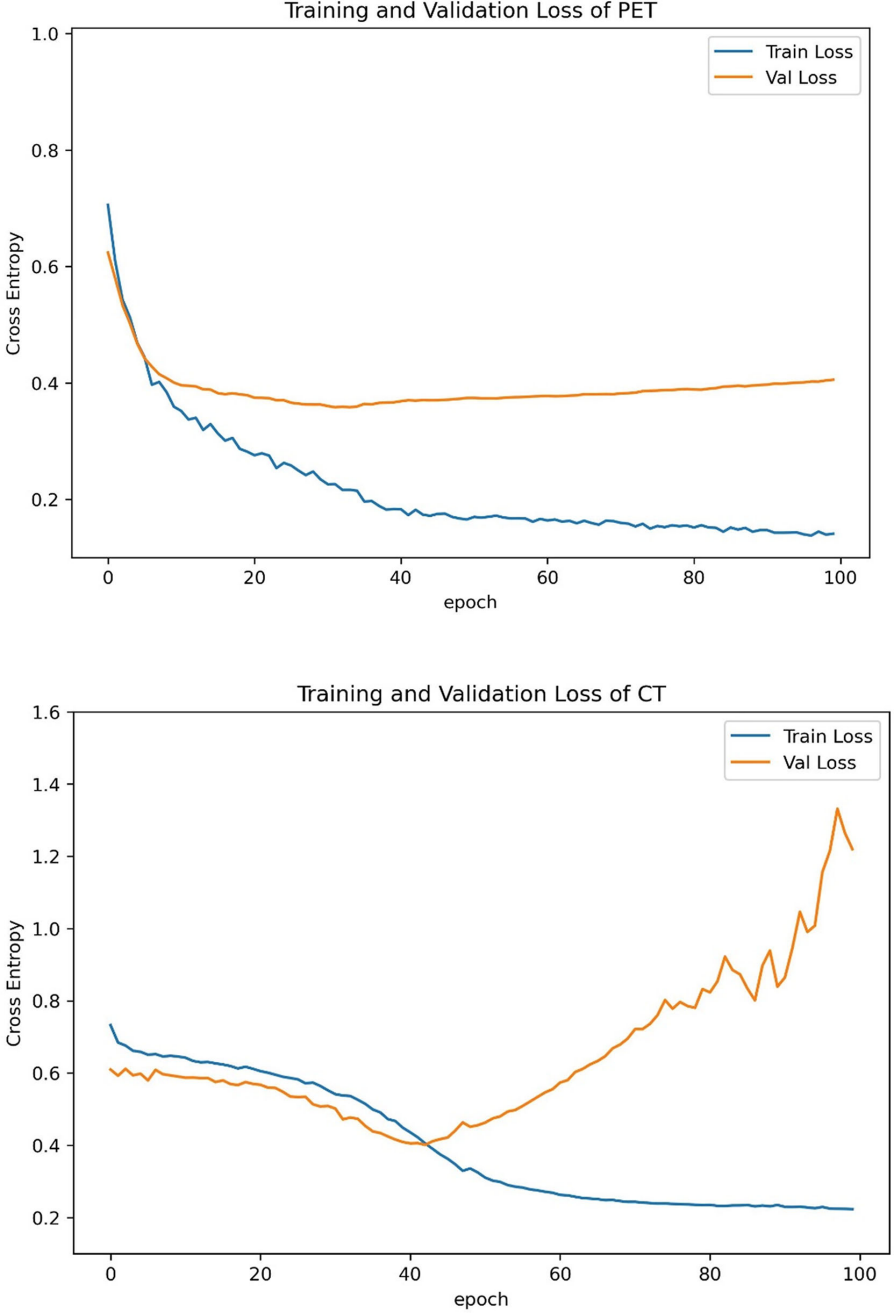
Training curves of PET and CT models. We stopped training at the 40th epoch because no further improvement can be gained in the validation loss.

The training curves of PET and CT are provided in **Figure 4**. The validation losses of PET and CT basically reached the minimum at 40~45 epochs, and then losses of training set and validation set estranged after 40 epochs. Therefore, we stopped training at the 40th epoch because no further improvement can be gained in the validation loss. The slowly decrease of validation losses suggests that the models have no overfitting before 45 epochs.

## Discussion

Recently, the therapeutic effect of limited surgery in patients with early-stage NSCLC without LNM has been proved to be significant, and limited surgery has more available lung tissue and lower perioperative mortality than standard treatment (28–30). Hence, there is an increasing need for accurately predicting OLM of cN0 LUAD before surgery in a non-invasive way.

Deep learning, which takes raw image pixels and corresponding class labels from image data as inputs and automatically learns representative information, has recently attracted much attention due to its excellent performance in image recognition tasks (17). In this study, we developed three deep learning models based on FDG PET/CT images for preoperative prediction of OLM in patients with cN0 LUAD. Our results presented that the complex model combining ^18^F-FDG PET and low-dose CT showed better diagnostic performances in distinguishing patients with OLMN and OLMP than either PET or CT alone.

Some studies demonstrated that CNN-based image analysis has been effectively applied in predicting LN status of lung cancer. For example, Zhong et al. (31) showed that a deep learning signature based on CT images could accurately predict occult N2 disease in patients with clinical stage I NSCLC. However, it is already known that PET/CT is more accurate than CT for direct assessment of LN status. Thus, confirming N0 status by CT is not enough. Tau et al. (32) demonstrated that using a CNN to analyze segmented primary tumors with PET in patients with pretreatment NSCLC can yield moderately high accuracy for designation of N category, but the use of segmented tumors as input data for the CNN was time-consuming and might affect the results. Moreover, most recent studies using deep learning (including the two studies discussed above) only performed single-modality analyses because integrating multimodal data is vulnerable to overfitting and poor generalization (22, 33). Wang et al. (34) mixed image patches of both modalities (PET and CT) into the same network, and the result showed that the performance of CNN was not significantly different from the best classical methods and human doctors for the classification of mediastinal LNM in patients with NSCLC. Such mixed setting may affect the final result because two different patches contained different types of diagnostic information. In this study, we processed the PET and CT patches with respective subnetworks and combined the results of the two different subnetworks at the output layers. For the internal validation set, the AUCs of the CNN in predicting nodal metastasis were as follows: ^18^F-FDG PET alone, 0.74; CT alone, 0.79; and 18F-FDG PET/CT, 0.81. For the prospective test set, the AUCs were as follows: 18F-FDG PET alone, 0.73; CT alone, 0.79; and ^18^F-FDG PET/CT, 0.87. Our results showed that the combined method, which makes full use of PET functional information and CT anatomic information, showed significantly great diagnostic performances in predicting OLM of LUAD.

A 2D CNN to discriminate between OLMN and OLMP in cN0 LUAD was successfully trained, validated, and tested in this study. Previous studies proposed that 3D CNN–based CT image analysis was used for classification in patients with lung cancer (23, 35). However, the increased complexity comes at a high computational cost. Another factor to consider is whether adding these interslice features would improve classification performance. Lee et al. (36) reported that a 2D CNN slice-based approach had better performance than 3D-CNN case-based approach for detecting intrapelvic tumor recurrence and metastases. The study of Vries et al. (21) also showed that the sagittal 2D CNN already performed with very high accuracy for discriminating between Aß-negative and -positive PET scans in patients with subjective cognitive decline. Therefore, we hypothesized that patients without a very large number of cases may be more applicable to 2D CNN architectures.

For clinical features, statistical analysis showed a significant difference in pathologic tumor size, CEA, and tumor SUVmax in the training set, which is consistent with our previous findings (16, 37). However, these clinical features were not all statistically significant in our validation and test sets, which may imply that the clinical utility of these features is limited.

There are several limitations to our current study. First, this was a single-center study with a relatively small sample size. Further improvement with multicenter and large-sample studies must be achieved before clinical use. Second, patients with multiple lesions were excluded because it is difficult to determine which lesion would cause OLM and should be input in the model. Therefore, predicting OLM of multifocal lung cancer needs to be further verified. Third, although statistical analysis of clinical data was performed, we did not integrate these clinical features into the deep learning model. Therefore, clinical parameters as another modality combined DL model should be studied in the future. Fourth, we did not use PET/CT fusion images because PET scan is difficult to rigidly match with CT scan in spatial location due to cardiac and respiratory motion artifacts. Last, limitation also obviously includes the opaque black box nature of the deep learning technology.

## Conclusions

We constructed a deep learning model that can successfully incorporate PET and CT images into a 2D CNN architecture to accurately predict OLM in patients with cN0 LUAD. Moreover, the deep learning model demonstrated a good predictive performance. This model may help to determine the patients who are eligible for limited resection.

## Data Availability Statement

The data analyzed in this study is subject to the following licenses/restrictions: The medical images are not publicly available due to the ethical considerations. The analysis code used in this study can be obtained by the corresponding author upon reasonable request. Requests to access these datasets should be directed to M-LO, 1427738937@qq.com.

## Ethics Statement

Informed consent from the retrospective patients was waived, and written informed consent was provided for patients in prospective test set.

## Author Contributions

R-XZ, Y-RW, L-DG, Y-QT, and M-LO collected the clinical information and the imaging data. R-XZ, Z-YZ, Y-GW, and M-LO were responsible for writing code and data analysis.

## Funding

This study was supported by Zhejiang Public Welfare Technology Application Research Project, China (LGF21H010009) and Wenzhou Science and Technology Program (no. Y20210222).

## Conflict of Interest

Author Y-GW was employed by General Electric (GE) Healthcare.

The remaining authors declare that the research was conducted in the absence of any commercial or financial relationships that could be construed as a potential conflict of interest.

## Publisher’s Note

All claims expressed in this article are solely those of the authors and do not necessarily represent those of their affiliated organizations, or those of the publisher, the editors and the reviewers. Any product that may be evaluated in this article, or claim that may be made by its manufacturer, is not guaranteed or endorsed by the publisher.
